# African-led health research and capacity building- is it working?

**DOI:** 10.1186/s12889-020-08875-3

**Published:** 2020-07-14

**Authors:** Victoria O. Kasprowicz, Denis Chopera, Kim Darley Waddilove, Mark A. Brockman, Jill Gilmour, Eric Hunter, William Kilembe, Etienne Karita, Simani Gaseitsiwe, Eduard J. Sanders, Thumbi Ndung’u

**Affiliations:** 1 Africa Health Research Institute, Durban, South Africa; 2 HIV Pathogenesis Programme, The Doris Duke Medical Research Institute, University of KwaZulu-Natal, Durban, South Africa; 3 Faculty of Health Sciences, Simon Fraser University, Burnaby, BC Canada; 4 Molecular Biology and Biochemistry, Simon Fraser University, Burnaby, BC Canada; 5 British Columbia Centre for Excellence in HIV/AIDS, Vancouver, BC Canada; 6 Imperial College London, London, UK; 7 Department of Pathology and Laboratory Medicine, Emory University, Atlanta, GA USA; 8 Rwanda Zambia Emory HIV Research Group, Zambia; Kigali, Rwanda and Emory University, Atlanta, USA; 9 Botswana-Harvard AIDS Institute Partnership, Gaborone, Botswana; 10 Department of Immunology & Infectious Diseases, Harvard T.H. Chan School of Public Health, Boston, MA USA; 11 Kenyan Medical Research Institute-Wellcome Trust Research Programme, Kilifi, Kenya; 12 Nuffield Department of Clinical Medicine, Centre for Clinical Vaccinology and Tropical Medicine, University of Oxford, Headington, UK; 13 Ragon Institute of MGH, MIT and Harvard University, Cambridge, MA USA; 14 Max Planck Institute for Infection Biology, Berlin, Germany; 15 Division of Infection and Immunity, University College London, London, UK

**Keywords:** Capacity building, Global health, Africa, Health research

## Abstract

**Background:**

Africa bears a disproportionately high burden of globally significant disease but has lagged in knowledge production to address its health challenges. In this contribution, we discuss the challenges and approaches to health research capacity strengthening in sub-Saharan Africa and propose that the recent shift to an African-led approach is the most optimal.

**Methods and findings:**

We introduce several capacity building approaches and recent achievements, explore why African-led research on the continent is a potentially paradigm-shifting and innovative approach, and discuss the advantages and challenges thereof. We reflect on the approaches used by the African Academy of Sciences (AAS)-funded Sub-Saharan African Network for TB/HIV Research Excellence (SANTHE) consortium as an example of an effective African-led science and capacity building programme. We recommend the following as crucial components of future efforts: 1. Directly empowering African-based researchers, 2. Offering quality training and career development opportunities to large numbers of junior African scientists and support staff, and 3. Effective information exchange and collaboration. Furthermore, we argue that long-term investment from international donors and increasing funding commitments from African governments and philanthropies will be needed to realise a critical mass of local capacity and to create and sustain world-class research hubs that will be conducive to address Africa’s intractable health challenges.

**Conclusions:**

Our experiences so far suggest that African-led research has the potential to overcome the vicious cycle of brain-drain and may ultimately lead to improvement of health and science-led economic transformation of Africa into a prosperous continent.

## Background

Africa comprises 15% of the world’s population but bears 25% of the global disease burden and produces only 2% of the world’s research output [1–3]. Significant challenges that contribute to this poor research output include: a dearth of well-trained and skilled researchers resulting in poor supervision of higher degree scholars, a lack of a critical mass of researchers even where pockets of excellence exist, weak or very limited progression pathways for those in scientific careers, and poor research infrastructure including a lack of access to scholarly tools such as scientific literature [4–8]*.* Support services that facilitate sustainable research are also often inadequate, such that academics and researchers on the continent work without the administrative, grant, financial, communication, and public engagement assistance that their counterparts enjoy in more resource-rich environments. These systemic problems facing researchers in Africa can often lead to ‘brain-drain’ and therefore perpetuation of inadequate training environments [5, 9, 10]. In addition, some challenges are external to Africa, but include legacies of colonialism, such as parachute researchers as well as funding and publishing structures that continue to favour Northern-based researchers [7, 10–13]. Can we turn the tide against this vicious cycle? What is the best approach to do this? Here, drawing on lessons from an African-led research and capacity building consortium, we discuss that African-led capacity building efforts on the continent are now the optimal way forward in order to achieve a long-lasting impact and highlight the importance of knowledge production by the global South [8, 10, 11, 14, 15].

It is encouraging that the past decade has seen a significant increase in research and capacity building investment on the African continent. Primarily, these investments have come from the global North but many African governments have also recently worked to increase local and regional financing for scientific research and capacity building, and this support is expected to grow as its importance is further appreciated [10, 12, 16, 17]. Historically, capacity-building programmes have taken a range of different approaches, e.g. strengthening North-South partnerships, strengthening national partnerships, and/or strengthening South-South partnerships. The result has been many funding initiatives and many consortia each with different levels of success and prospects for sustainability [6, 10, 18–24]*.* Overall, these efforts have greatly contributed to increasing scientific research capacity on the continent, particularly in relatively ‘quick yield’ disciplines such as epidemiology and research ethics strengthening, and have helped to develop a firm foundation that can now be built on (although research capacity across the continent is uneven) [6, 8, 10, 22]. However, most efforts in Africa to date have not only been financially supported but also driven - both at the official programme level and at the level of individual scientists– from outside the continent [10, 22]. This does not augur well for the development of sustainable African-led knowledge production pipelines that might lead to local economic benefits or sustainability of these activities within local socioeconomic frameworks [8, 15, 25].

## The benefits of African-led efforts

Recently, major international funders have appreciated the importance of shifting internationally-led research and capacity building efforts towards more African-led models [6, 18, 24, 26]. Two specific examples of recent African-led funding initiatives include: 1) US $135 million through the Medical Education Partnership Initiative (MEPI) from 2010 to 2015 (supported by the United States’ National Institutes of Health (NIH) and the U.S. President’s Emergency Plan for AIDS Relief (PEPFAR]); and 2) a commitment of approximately $180 million through the Human Heredity and Health in Africa (H3Africa) initiative from 2011 to 2021 (supported by the NIH, the Wellcome Trust and the African Academy of Sciences). The reality is that Africans are often, although not always, best placed to identify and contextualise the most relevant and pressing local problems, which should inform the development of national and international partnerships tasked with originating and leading research agendas [7, 9, 10, 15, 22]. We appreciate that external support and collaboration is still essential, and that some challenges do not necessarily need enhanced research but simply political will to solve them (e.g. insufficient childhood vaccinations, inclusive community engagement, and affordable quality primary and secondary education) [6, 27]. However, we and others believe that scientific health research efforts and capacity on the continent will not further improve, or become sustainable, without Africans increasingly taking a leading role [8, 15].

The immediate benefits for African researchers are enhanced local ownership of activities and new opportunities for steady and sustained skills building of staff and trainees, which will hopefully lead to improved research outputs, including African-led first and senior author publications and grants awarded to African researchers [10]. When research is led by African scientists, not only may more locally relevant topics be targeted, but it may be more likely that study findings will be communicated by African researchers in a cultural and policy context that is more accessible and relevant to local populations [8, 15, 28]. This is one reason we believe that recommendations from African-led studies may resonate better and have better uptake among African policy makers compared to research results produced by teams that are largely internationally-led. In addition, African-led research will further increase opportunities for senior African scientists to act as role models for junior scientists (e.g. to advise on how to successfully navigate university administrative systems), increase visibility of African scientists, facilitate South-South collaborations, and strengthen African institutions [15, 29]. While external collaboration, knowledge exchange and financial support is critical to capacity building in Africa, and there are many tangible immediate benefits to existing collaborations, sustainable scientific development efforts on the continent will only happen if research and capacity building are led, managed and owned by its citizens [10]. Table 1 outlines the key immediate benefits of African-led scientific capacity building efforts on the continent.

**Table 1:** Key benefits of African-led capacity building

Research more aligned to and addressing key local scientific and health challenges	
Enhanced local ownership of activities	
New opportunities for skills building and developing of local scientists and staff	
Increased and improved research outputs including more first and last author papers	
Recommendations from African-led studies may resonate more and lead to better uptake by local policy makers	
More opportunities for senior African scientists to act as role models	

In 2015, the Wellcome Trust launched the DELTAS Africa initiative, in collaboration with the UK Department for International Development (DFID) and the Bill & Melinda Gates Foundation. DELTAS Africa is currently supporting 11 research consortia across Africa with an investment of over $100 million USD over an initial period from 2015 to 2020, making it one of the most ambitious initiatives by a major international donor in biomedical sciences to channel significant funding to African-led research teams. The key innovation of this initiative is that the Wellcome Trust has handed control and ownership to the African Academy of Sciences (AAS)‘s Alliance for Accelerating Excellence in Science in Africa (AESA), which is headquartered in Nairobi, Kenya, and is also supported by the African Union Development Agency (AUDA-NEPAD). The aim here is to base all aspects of African science and capacity building efforts on the continent, from conceptualisation of ideas, the implementation of the grant, to evaluation and management of the programme as a whole. The DELTAS Africa initiative is currently in its last year of a 5-year funding cycle and the Phase II call for proposals has been announced (for a 2021–2025 cycle). From the observations and feedback to date it is clear that the DELTAS Africa initiative is likely to be one of the most impactful efforts ever in terms of African research production, numbers and quality of African trainees, and strengthening of African institutions, particularly with respect to knowledge translation and community and public engagement (CPE). As leaders of the Sub-Saharan African Network for TB/HIV Research Excellence (SANTHE), one of the consortia funded by the initiative, we hereby reflect on the approaches used by our consortium and share why we have been successful, in addition to other learnings from our efforts as an African- led science and capacity building programme.

## The Sub-Saharan African Network for TB/HIV Research Excellence (SANTHE)

**Fig. 1:**
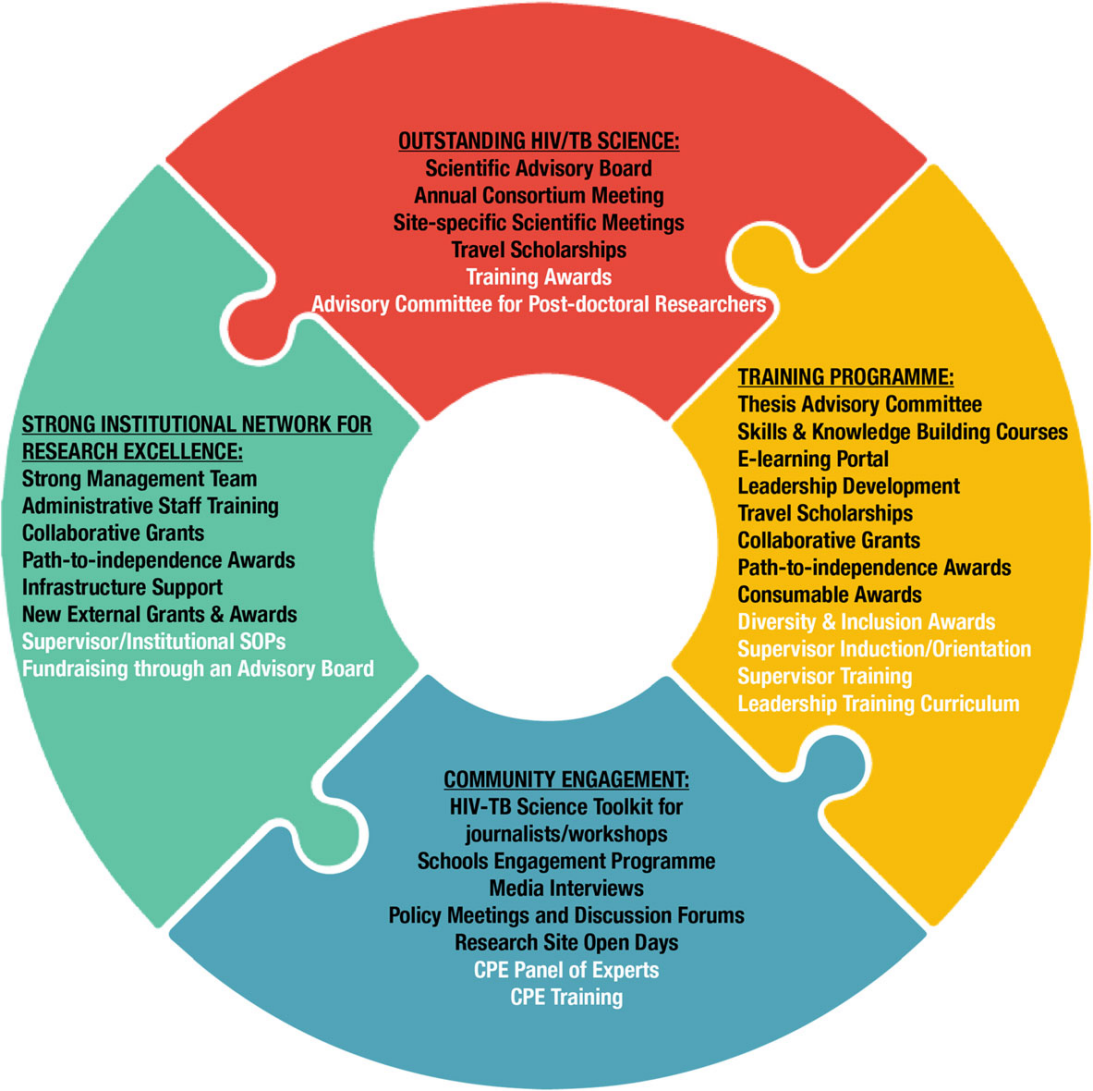
Outline of key tools used in the SANTHE consortium to support scientific capacity building. Tools listed in black font were utilised in the first 5 years of our consortium. Highlighted in white font are new tools, identified through monitoring and evaluation efforts, that we feel would enhance our programme in the future. CPE = Community and Public Engagement. SOP = Standard Operating Procedures

SANTHE [https://www.santheafrica.org/] is an African-led HIV and TB research and capacity building consortium (currently based at five primary sites in South Africa, Botswana, Kenya, Rwanda and Zambia) which received an investment of approximately $11.2 million USD over 5 years. We aim to carry out cutting-edge HIV/TB research, train the future leaders of African science, develop strong institutional networks to facilitate research on the continent, and facilitate effective CPE to ensure meaningful research translation and maximum community impact. The goals of institutional strengthening and CPE cannot be overemphasised - they are as critical to the long-term sustainability of the DELTAS funded programmes as are our scientific activities and capacity development of our researchers. We believe that these efforts should be led and executed by locally-based individuals who have in-depth understanding of the institutions and communities who stand to benefit from the research. From 2015 to the present, SANTHE has established a strong foundation with, as an example, 119 peer reviewed publications in the past 4 years. We have demonstrated a good level of success in supporting our Fellows to convert their scientific findings into manuscripts with 42 of these publications first-authored by SANTHE Fellows. We recruited 105 trainees from 10 different African countries who included 15 graduate interns, 38 Masters students, 38 PhD students and 14 post-doctoral researchers with 64% of these being females. To date, 42 have completed their Fellowships (which includes 17 Masters graduates, 3 PhD graduates, and 4 individuals who have upgraded from Masters to PhD). Our Fellows have given 82 presentations at international conferences which include CROI, IAS and Keystone. Figure 1 outlines the key tools that were introduced initially to support our scientific capacity building efforts. As an example, to support the production of outstanding science we convened a scientific advisory board of outstanding scientists familiar with the state of science and training institutions in Africa to provide scientific and capacity building advice to the consortium. This group of individuals met yearly at our annual consortium meeting (ACM) and were consulted to provide feedback on an ongoing basis. We have provided continuous and systematic monitoring and evaluation of the effectiveness of our interventions and used our data to help propose new solutions or amend existing ones. As we look towards SANTHE’s future, and a potential expansion to 3 new partner sites (in Cameroon, Zimbabwe and Uganda), we have assessed the tools we use, with help from internal and external monitoring and evaluation efforts, and propose to introduce new tools to supplement our initial offering (highlighted in Fig. 1). The new tools include the development of a leadership development curriculum, intensive and holistic supervisor training, the incorporation of a panel of experts to help guide our CPE efforts, and the provision of an advisory committee for post-doctoral researchers.

**Fig. 2:**
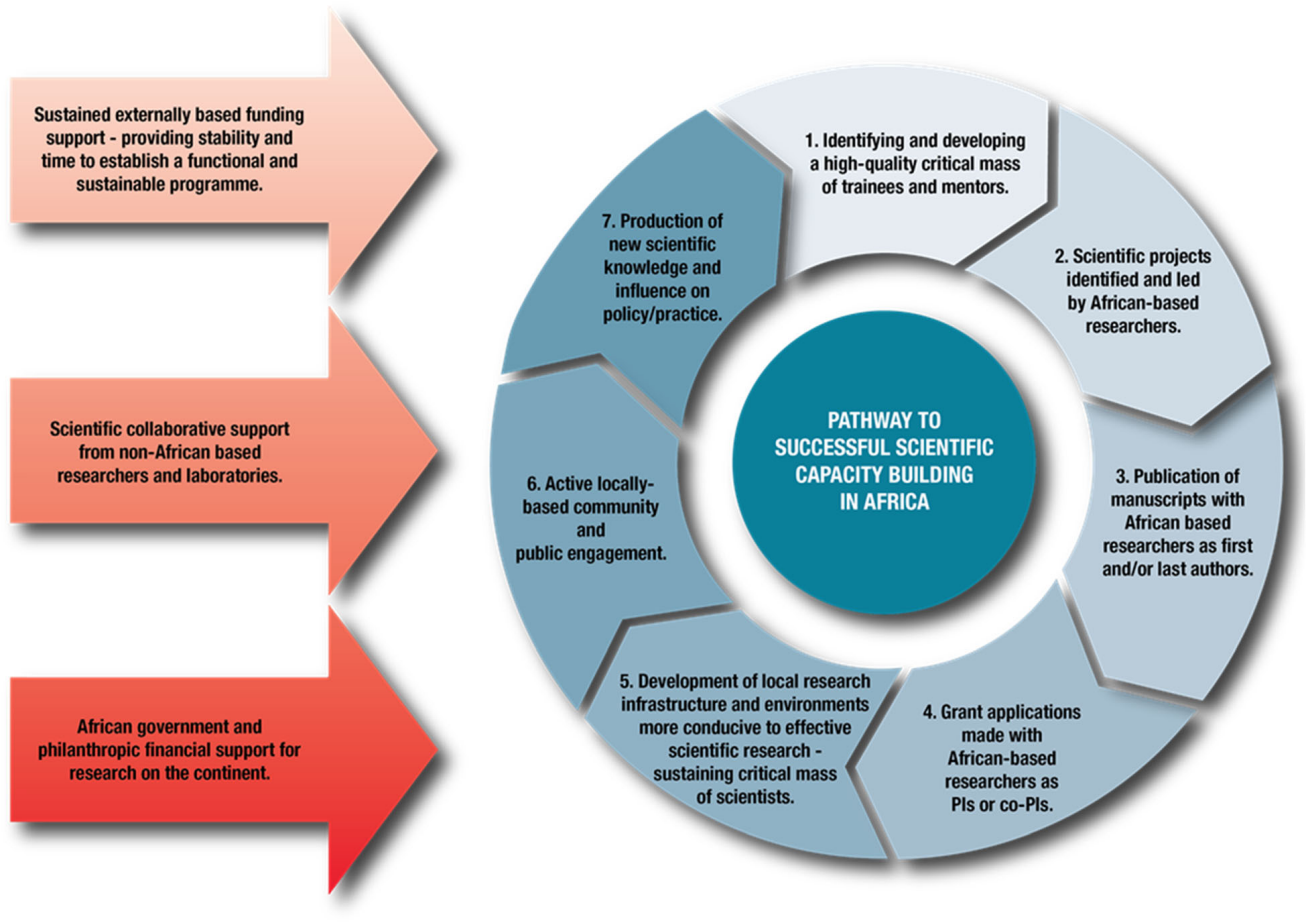
Overview of our proposed pathway to successful scientific capacity building in Africa. Based on our experiences, we highlight critical elements for scientific capacity building in Africa and also note the need for an evolution of health research funding from the current overreliance on external funding towards Africa-based funding

Our proposed pathway for successful capacity building in Africa is highlighted in Fig. 2. Step 1 is the identification and development of a critical mass of trainees and supervisors. This results in an increasing number of scientific projects identified and led by African-based researchers, which in turn will lead to publications with African-based scientists as first and last authors and increasing numbers of grant applications with African-based scientists as principal investigators (PIs). Increased local grant funding, infrastructure development, and investment in training of science support staff will lead to the development of local research environments optimally supporting ongoing science and the scientists based therein. The foundation of an empowered cadre of local scientists, trainees and support staff will then lead to locally-driven CPE efforts that are dovetailed with relevant cultural and policy contexts applicable to African settings. This combination of a rich and growing pipeline of scientific expertise, research support and CPE activities will then lead to more efficient production of new knowledge and translation into policy and practice. Our current model acknowledges that sustained external funding support will initially be needed, providing time and stability to establish a functional and sustainable programme. External funding will need to be combined with scientific collaborative support from non-African based researchers and laboratories. However, over time increasing levels of African government and philanthropic financial support for research on the continent hold the key to unlocking Africa’s scientific potential. While we acknowledge that each country and consortium may have their own unique challenges and require their own tools, based on our experiences we highly recommend the following key components (and associated tools) to optimally support the capacity building pathway:** 1.Directly empowering African-based researchers, 2. Offering quality training to large numbers of junior and early career African scientists and support staff, and 3. Effective information exchange and collaboration.**

### Directly empowering African-based researchers

Our first step towards directly empowering African-based researchers involved the identification and support of locally initiated and led efforts. The scientific concept sheets for proposed studies funded by SANTHE were developed by supervisors who are mostly based on the continent and peer-vetted (by a team of three scientists based at the consortium secretariat site in South Africa, or invited experts affiliated with the consortium), for relevance to local problems, feasibility of implementation and potential impact. Trainees are identified to work on selected projects following innovative and competitive recruitment procedures (that include panel interviews, critique of an assigned manuscript, a written scientific proposal assignment, and online abstract and numerical reasoning tests). SANTHE makes stipend and consumable support available for these projects thus benefiting trainees and their supervisors, particularly more junior supervisors with worthy ideas but limited grant funding support. Although SANTHE benefits from non-African collaborating partners for scientific input, collaboration, and assistance with training of Fellows, projects are mainly conceived and executed by African-based supervisors. Research is performed primarily in Africa, with students enrolled in both African institutions of higher learning, and in universities in well-resourced countries that are affiliated with SANTHE (if local facilities are not yet adequate). Our second step towards directly empowering African-based researchers involved the inclusion of all our scientists in a wide network to support scientific efforts. As an example of one of our tools, our ACM has proved to be highly valued by both our junior and senior scientists, and in addition to providing an opportunity to provide scientific critique and obtain feedback on ongoing science, has allowed the growth of training and scientific collaborative efforts, allowing the expansion of our research focus and enhancing our ability to tackle projects that would not be possible if each site operated in isolation.

One advantage of holding such a large and diverse capacity building grant is that it makes it possible to support initiatives that would normally be difficult to fund on their own, due in part to limited funding options on the African continent, such as career advancement opportunities including our Path-To-Independence Awards and our Collaborative Grants. Between 2015 and 2020 SANTHE funded 15 Collaborative Grants (up to $50,000 USD each). Awards were generally given to junior investigators and, involve researchers from a total of 23 different African sites. SANTHE has also awarded five Path-to-Independence (PTI) awards ($100,00 USD each), which provide critical bridge funding to support young scientists who are establishing themselves as independent investigators (often a critical bottleneck in a scientist’s career development pathway). As an example, one PTI award enabled a former SANTHE post-doctoral researcher to return to his home country of Cameroon after spending eight years training in South Africa. In addition to establishing his own research programme, this award facilitated new collaborations and expanded the SANTHE consortium to include a second site in Francophone Africa. It is of great satisfaction to us that many of our early career scientists who received SANTHE seed funding have leveraged these opportunities to benefit from additional funding including the AAS’s FLAIR Fellowships and Wellcome Trust Fellowships. Overall, the investment that SANTHE received has directly benefited African-based researchers by raising their profile and that of their institutions, and enabling them to directly identify and address problems, set a research agenda, supervise students on these projects on site, promote their careers in the process, and directly impact local and international stakeholders. Crucially, one demonstration of the scientific impact of these efforts is that important discovery and policy impacting research has been performed within a short period of time [30–32].

### Offering high quality training to large numbers of junior African scientists and scientific support staff

SANTHE aspires to provide high-quality training for a large cohort of African trainees so that graduates are internationally competitive. It is anticipated that these efforts will help to reduce ‘brain-drain’, which is more likely to occur when African researchers are trained primarily overseas. The emphasis on large numbers, although variously defined is deliberate because it should be noted that funding through our consortium is not only building the capacity of individual researchers but also directly contributing to the building of a critical mass within institutions. Instead of identifying single talented individuals, consortium-level funding is able to invest in providing student stipends and research support to enable recruitment of a cohort of trainees at a site, providing an opportunity for peer support and interactions that are critical to sustaining research interest, increasing the numbers of scientists at one site, and helping to avoid the frequently encountered challenge of working in relative intellectual isolation.

One critical component of high-quality training we have emphasised is skills development. We have provided training by developing in-house value-adding courses or workshops and partnering with key collaborators, including academic supervisors. For example, we have offered internal manuscript and grant writing workshops and partnered with institutions such as Simon Fraser University and the Ragon Institute of MGH, MIT and Harvard to offer specialised courses that have included international faculty and trainees. This has proved to be a cost-effective approach, facilitated exploration of cutting-edge topics and approaches while allowing scientists beyond our network to benefit from the SANTHE funding. Another example of one such course took place in January 2019 when SANTHE co-hosted a workshop on HIV reservoirs and evolution (in collaboration with the Max Planck Society and with additional financial support from the Victor Daitz Foundation, a South African philanthropy), which brought together leading researchers in the field of HIV cure research and treatment failure and provided SANTHE scientists the opportunity to learn about the latest developments in research in this area and engage with other leading scientists. Examples of other course topics include CPE, biostatistics, phylogenetics, and immunology. In all our efforts we work to support the development of independent and critical thinking.

Travel scholarships are another tool that we used to enhance training, exposure and representation of our scientists and their work, enabling our trainees to attend conferences and key training events, and supporting the production of outstanding science. Travel scholarships have allowed us to support trainees to attend external training events and acquire training from other laboratories and sites. This support for our trainees to visit institutions in well-resourced countries (or to other African research sites) to learn new skills and to exchange ideas with international colleagues is highly beneficial. Increasingly, travel funding is being utilized for knowledge exchange and training within the network and amongst sub-Saharan African sites. For example, one of our PhD Fellows based at the Centre de Recherche sur les Maladies Emergentes et Re-Emergentes (CREMER) is conducting aspects of his research at the Africa Health Research Institute (AHRI) in South Africa, and will return to Cameroon with the necessary molecular biology skills to continue his research. By managing the process through African-based investigators and from African institutions, we believe that we can better match trainees with local needs and offer more conducive environments to integrate new knowledge into the core experience of African-led research teams. Over the last five years we have awarded 205 Travel Scholarships (awards ranged from $162 USD to $18,536 USD (average of $3256 USD)). 15% of awards supported training in other labs, 17% were for conference attendance and 68% were used to attend workshops/courses (both internal and external).

A second component in offering high-quality training to our junior scientists involves the promotion of effective mentorship. SANTHE has benefited from a large pool of scientists based in both well-resourced countries and local institutions. These individuals have acted as supervisors to our Fellows, as members of our SAB, or as members of our Thesis Advisory Committees (TAC). As an example, we have 74 supervisors representing 34 institutions (21 of which are based in Africa). We designed and introduced evaluation forms to enable both trainees and supervisors to provide feedback on the ongoing supervision and project progress**.** Our supervisors and postgraduate trainees have benefited from the introduction of TACs, as demonstrated by the very positive feedback received in these evaluations. These committees, which are not a requirement in most African institutions of higher learning, consist of at least three individuals, one of which is the primary supervisor to the Fellow. We have also identified the need for similar formal support for postdoctoral researchers and early career researchers, and will introduce this in the next phase of our programme. Indirect scientific and career mentorship has also been available through access to the larger SANTHE community. Furthermore, we plan to introduce comprehensive and holistic supervisor training on topics from conflict resolution and effective supervision techniques to core skills training such as biostatistics and CPE.

Our third component of high-quality training for our scientists involves leadership and career development support. Examples of this include soft skills training and opportunities to take leadership roles in SANTHE events and at an institutional level. We are currently further developing our curriculum to include compulsory training on topics from grants management to comprehensive CPE skills. These trainees also receive support through direct investment in our research environments, yet another advantage of receiving consortium-level funding. Our training has not just focused on our scientists; we have also funded support staff training in the areas of leadership, administration, grant management, financial management, and science communication. One key reason for SANTHE’s success as a consortium has been its strong secretariat (five full time staff members providing comprehensive logistical support in key science support areas). The advantage of this support being African-led is that it has enabled us to learn and build capacity in these key areas and therefore allow the strengthening of the research environments at all our sites. We have also taken the lead and helped to train others beyond our immediate SANTHE community. This is demonstrated in part by our leadership in activities such as the three-day Risk Management Workshop at the DELTAS 2018 Annual Meeting.

### Effective information exchange and collaboration

A pervasive challenge to scientific excellence in Africa is the lack of opportunities to meaningfully engage with peers and experts in one’s field of research. It is vital for scientists to have opportunities to exchange ideas and information, to obtain critical feedback on their work, and to partner on projects to facilitate the ability to address cutting-edge issues. SANTHE has worked hard to create a forum for the exchange of ideas and interactions between researchers both on the African continent and beyond. For example, in addition to our ACM, through our site-specific research days (at which all SANTHE trainees at each site get an opportunity to give an oral presentation of their ongoing research and receive detailed feedback and critique), and our monthly meetings (opportunities for trainees to present their scientific or CPE activities). We have also hosted seminars, workshops and roundtable discussions to share and discuss information, results, and key challenges.

Another step towards effective capacity building is the development of strong collaborations in key areas including science, training, science support and community and public engagement. Effective cross-site collaborations are vital in helping to address scientific questions as a consortium, catalysing research synergies. To help assist with this with have used our collaborative grants and travel scholarships as key tools. A key part of our efforts is sharing information beyond SANTHE and regularly take the lead in activities to encourage dialogue between researchers even beyond the continent. Examples of our interaction with the global scientific community include hosting symposia and satellite sessions at international conferences. For example, the Strategies for Diagnosing and Managing Acute HIV Infection in the Context of PrEP and Immediate ART Symposium at IAS-AIDS in 2018 [33]. Our efforts to collaborate extend beyond the merely scientific to include all areas of science support. For example, in 2019 we hosted the 2019 Africa Asia Communications Forum which aims to identify ways to maximise impact from research being performed, in turn supporting the development of stronger communication and public engagement activities. This is an important component of our efforts to support the promotion of African science.

## Challenges

Other DELTAS programmes have reported similar levels of success, e.g. publications, grants, new degree programmes, new departments, new and improved infrastructure, and new lines of African-led research. It has been observed that the success of African-led capacity building is often linked to strong programme directors [33, 34]. Interestingly, key players of these new initiatives are often themselves former trainees of past programmes, whether locally or internationally-led, which is highlighted by the NIH’s Fogarty Programme [35]. This indicates that the success of current African-led research is, in reality, a consequence of past programmes bearing fruit, with the added benefit that the current programmes are likely to have a wider impact in terms of numbers, sustainability and creation of thriving hubs of research that may support new career opportunities for African scientists [10].

However, despite the reported success there are many challenges. SANTHE has experienced numerous teething problems which include the difficulties of running a large-sized consortium, examples of which include: difficulty in implementing interventions easily across all sites due to diversity in site capacity or ethos of institutions e.g. variable degree requirements; non-electronic communication issues; balancing merit and equity in funding allocations; and the sustainability of consortia-level funding. Some struggles SANTHE has faced are representative of general capacity inadequacies and inequities on the continent, for example: difficulty in identifying large numbers of high-quality trainees for the positions available; the sustainability of trainee research careers; navigating university-level bureaucracy; and training students at research institutions versus universities. Others have also reported issues that include: not enough research-protected time for faculty-level researchers; too many trainees per supervisor; lack of funding schemes catering to the increased numbers of trainees as they progress in their careers; internet speed issues; lack of local degree training programmes at universities; academia viewed as an unrealistic career path; insufficient funding for research projects; and pipeline issues for high-quality trainee recruitment e.g. poor training at school and undergraduate level [5, 7, 9, 19, 22, 26, 36].

As a result of the learning we have experienced over the last 5 years, there are a number of key changes we wish to implement. One example of this, building on our findings from our recent Wellcome Trust Diversity and Inclusion Award, is the introduction of Diversity and Inclusion Awards (DAIA). One example is a DAIA provided to a female trainee that enabled her to bring her 10-year-old daughter with her from Kenya to South Africa so that she could attend the SANTHE Biostatistics Course. The course took place during the school holiday period and the trainee, a single mother, had no other suitable child-care available during this period and would therefore have been forced to forgo the course. It is clear that there are often child-related barriers preventing our scientists from attending key events and through this grant, we are now in a position to assist with these challenges and in doing so are helping support all our scientists as they progress in their careers.

Although these challenges can be frustrating, we at SANTHE have gained invaluable experiences and insights through a ‘trial and error’ process on some aspects of our award. These experiences are worth sharing and learning from, since they are undoubtedly not confined or unique to SANTHE. Although there have been efforts in the past, now more than ever there is a need to explore how the lessons learnt through health research capacity building initiatives can be shared and harnessed most effectively to improve the design, evaluation, cost effectiveness and overall impact of our efforts [5, 12, 14, 24, 27, 34, 37–39]. This knowledge sharing could continue through an interactive meeting or conference (with representatives from key stakeholders in capacity building initiatives in Africa, such as funders, government representatives, university leadership and trainees), where the participants can present best practices, lessons learned, and ways to allocate resources more efficiently for greater impact. Through such efforts, we may be able to identify and prioritise the training and capacity needs going forward and discuss how best to address them with available resources. This in turn will improve leadership and scientific output on the African continent and beyond. A valuable contribution to this process will be the findings of the DELTAS Africa Learning Research Programme (LRP) which aims to produce research-based learning from the DELTAS Africa initiative and investigate how best to train and develop world-class researchers and promote research uptake.

## Conclusions

In conclusion, we believe the following are key target areas required to support the pathway for successful capacity building in Africa: **1.Directly empowering African-based researchers, 2. Offering quality training to large numbers of junior African scientists and support staff, and 3. Effective information exchange and collaboration.** Despite the clear advantages and success of African-led capacity building, continued and sustainable funding support is still necessary. There are still many challenges to overcome. The current programmes will need to be evaluated and assessed but it is important that a long-term view in support of sustaining the programmes be promoted. Alternative funding mechanisms, such as through local tax-payers and local philanthropies must also be encouraged. Although increased investment in science by African governments is being made, sub-Saharan Africa still lags behind other global regions with countries, on average, spending 0.4% of GDP on research and experimental development (R and D) (contrasting with a 2.5% by North America and Western Europe and a target of 1% of GDP invested on R and D which was set by the African Union) [17, 18]*.* However, some countries, including South Africa and Kenya, are approaching this 2025 target and are currently investing around 0.8% of GDP. Increased domestic investment in research is needed to accelerate the long-term health and development progress required to meet the Sustainable Development Goals by 2030. It is clear that long-term investment from international donors and increasing funding commitments from African governments and philanthropies will be needed to realise a critical mass of local capacity and to create and sustain world-class research hubs that will be conducive to address Africa’s intractable health challenges. African-led research must eventually translate into African-funded research. At the moment, what is clear is that the tide is turning and scientific capacity building on the continent is working. The current efforts must be seen to their logical end- which is to see a science-led health and economic transformation of Africa into a prosperous continent.

## Data Availability

N/A

## Abbreviations

HIVR4P: HIV Research for Prevention Conference
MEPI: Medical Education Partnership Initiative
NIH: National Institutes of Health
PEPFAR: U.S. President’s Emergency Plan for AIDS Relief
H3Africa: Human Heredity and Health in Africa
DFID: UK Department for International Development
AAS: African Academy of Sciences
NEPAD Agency: New Partnership for Africa’s Development Planning and Coordinating Agency
SANTHE: Sub-Saharan African Network for TB/HIV Research Excellence
PTI awards: Path-to-Independence
LRP: DELTAS Learning Research Programme
R and D: Research and experimental development
